# Mitochondrial Dysfunction in Chronic Respiratory Diseases: Implications for the Pathogenesis and Potential Therapeutics

**DOI:** 10.1155/2021/5188306

**Published:** 2021-07-27

**Authors:** Wen-cheng Zhou, Jiao Qu, Sheng-yang Xie, Yang Sun, Hong-wei Yao

**Affiliations:** ^1^Department of Pharmacy, The First Affiliated Hospital of Zhejiang Chinese Medical University (Zhejiang Provincial Hospital of Traditional Chinese Medicine), Hangzhou 310006, China; ^2^State Key Laboratory of Pharmaceutical Biotechnology, Department of Biotechnology and Pharmaceutical Sciences, School of Life Science, Nanjing University, Nanjing 210023, China; ^3^School of Pharmacy, Anhui Medical University, Hefei 230032, China

## Abstract

Mitochondria are indispensable for energy metabolism and cell signaling. Mitochondrial homeostasis is sustained with stabilization of mitochondrial membrane potential, balance of mitochondrial calcium, integrity of mitochondrial DNA, and timely clearance of damaged mitochondria via mitophagy. Mitochondrial dysfunction is featured by increased generation of mitochondrial reactive oxygen species, reduced mitochondrial membrane potential, mitochondrial calcium imbalance, mitochondrial DNA damage, and abnormal mitophagy. Accumulating evidence indicates that mitochondrial dysregulation causes oxidative stress, inflammasome activation, apoptosis, senescence, and metabolic reprogramming. All these cellular processes participate in the pathogenesis and progression of chronic respiratory diseases, including chronic obstructive pulmonary disease, pulmonary fibrosis, and asthma. In this review, we provide a comprehensive and updated overview of the impact of mitochondrial dysfunction on cellular processes involved in the development of these respiratory diseases. This not only implicates mechanisms of mitochondrial dysfunction for the pathogenesis of chronic lung diseases but also provides potential therapeutic approaches for these diseases by targeting dysfunctional mitochondria.

## 1. Introduction

**Figure 1:**
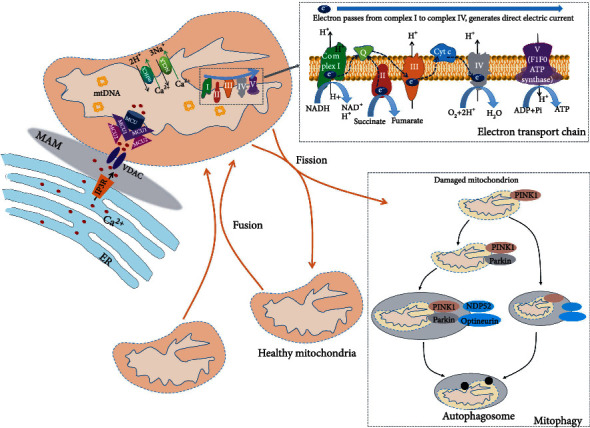
Normal mitochondria are sustained by stabilization of MMP, balance of mitochondrial calcium, integrity of mtDNA, and timely clearance of damaged mitochondria via mitophagy. The electron flows from complex I to complex IV, which creates a direct electric current that terminates in subunit II of complex IV for MMP. This is accompanied by the synthesis of ATP. Mitochondria are closely linked to the ER. They intimately and dynamically communicate, thereby forming very dynamic platforms termed MAM. The delivery of ER-mitochondria calcium mainly depends on IP3 receptors (IP3R) and voltage-dependent anion channel (VDAC). Mitochondrial calcium uptake is mediated by the MCU complex, while calcium release depends on mHCX and NCLX systems. Mitochondrion harbors its own DNA, which encodes many critical proteins for the assembly and activity of mitochondrial respiratory complexes. Mitochondria are dynamic through fission and fusion. Mitophagy is a selective and adaptive response and plays a critical role in surveilling mitochondrial quality and preventing accumulation of dysfunctional mitochondria.

Mitochondria are subcellular organelles that originate from a bacterial symbiont, containing two separate and functionally distinct outer and inner membranes that separate the intermembrane space and matrix compartment [1, 2]. It is considered the powerhouse of cells with energy generation in the forms of adenosine 5′-triphosphate (ATP) via oxidative phosphorylation (OXPHOS) on the electron transport chain (ETC) [3]. Mitochondria differ from other organelles and have their own maternally inherited DNA (mtDNA) [4]. mtDNA contains 37 genes coded for two ribosomal RNAs, 22 transfer RNAs, and the remainder 13 encoding proteins involved in OXPHOS. The mechanism of mtDNA replication is complex, which involves several critical enzymes, including the heterodimer mtDNA polymerase, DNA helicase Twinkle, mitochondrial RNA polymerase, mitochondrial single-strand DNA-binding protein, topoisomerase 3, and DNA ligase III [5–7]. Existing studies reveal that mtDNA replication is critically important to mitochondrial biogenesis [8]. The latter is a process of growth and division of preexisting mitochondria rather than de novo synthesis, depending on the coordination of both mitochondrial and nuclear genomes [9]. Mitochondrial numbers are regulated by mitochondrial biogenesis to meet the energy demands of the cell, which process is associated with mitochondrial fusion and fission. Fission causes additional smaller mitochondria, which promotes mitophagy, cell proliferation, and reactive oxygen species (ROS) production. Fusion results in the interconnection of the mitochondrial network as well as communication with endoplasmic reticulum (ER) and diffusion of matrix content among mitochondria, which dilutes accumulated mtDNA mutations and oxidized proteins [9, 10]. At the molecular levels, both optic atrophy 1 at the inner membrane and mitofusin 1 and 2 at the outer membrane mediate mitochondrial fusion. Fission is controlled by dynamin-related protein 1 and its docking proteins, mitochondrial fission protein 1, mitochondrial fission factor, and mitochondrial elongation factors 1 and 2 [11, 12]. In addition, mitophagy is an important control system for the selective elimination of damaged mitochondria. Furthermore, the close apposition between the ER and mitochondria is conducive to intimately communicate, forming very dynamic platforms termed mitochondria-associated membranes (MAM), which facilitate calcium flux into the mitochondria. Mitochondrial calcium flux participates in regulating essential functions, including metabolism, energy production, and apoptosis [13]. Hence, maintaining mitochondrial function and homeostasis is essential for healthy cellular function and survival. Mitochondrial homeostasis is associated with stabilization of mitochondrial membrane potential (MMP), balance of mitochondrial calcium, integrity of mtDNA, and timely clearance of damaged mitochondria via mitophagy (Figure 1). Once mitochondrial homeostasis is disrupted, it can disturb the OXPHOS process and induce accumulation and generation of side products, including ROS, which further cause mitochondrial dysfunction, forming a vicious cycle harmful to cell function [14].

**Figure 2:**
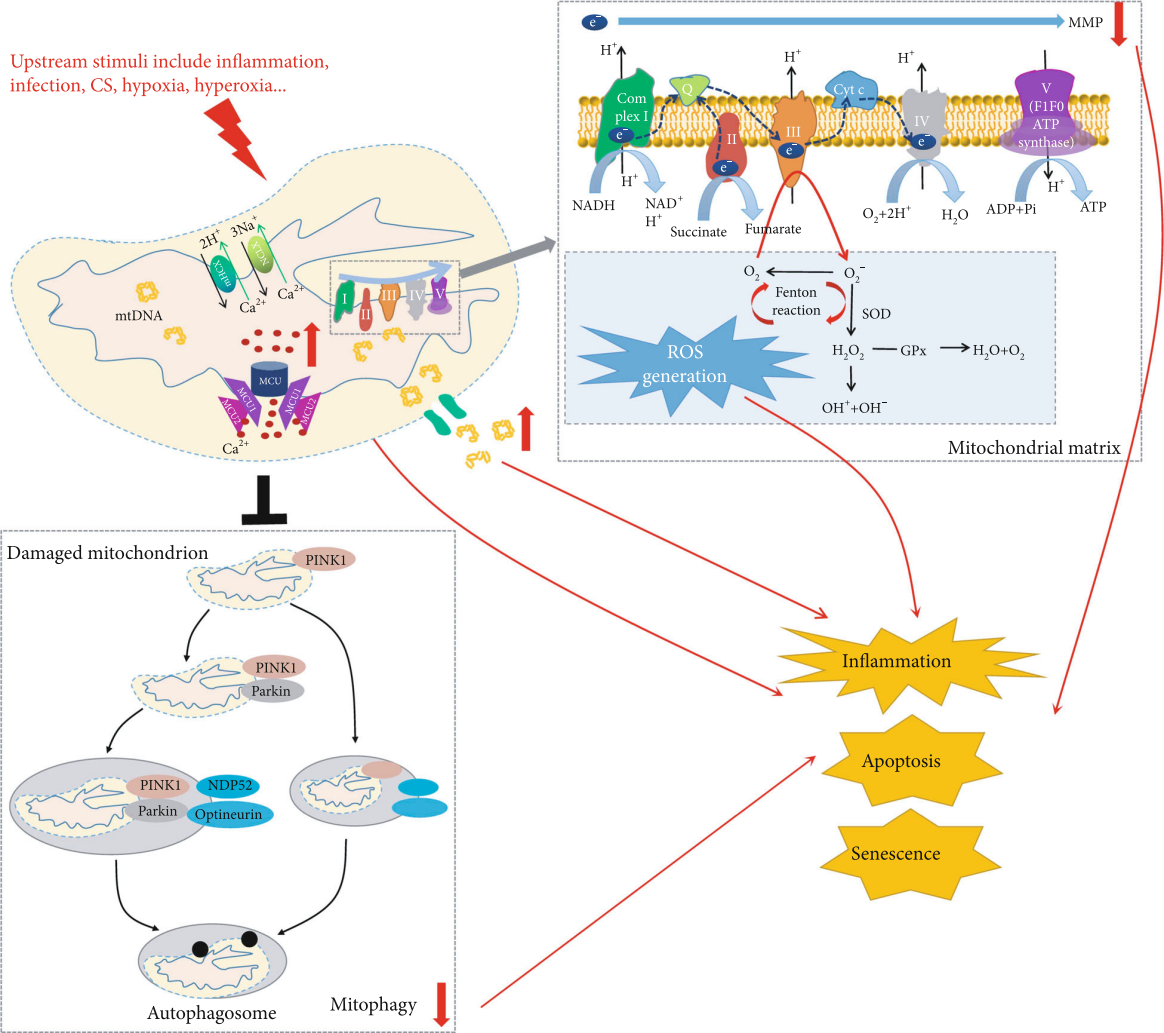
Mitochondrial dysfunction is characterized by increased mtROS, reduced MMP, increased calcium influx, mtDNA damage and release, and abnormal mitophagy. Mitochondrial homeostasis can be disrupted by upstream stimuli including inflammation, infection, CS, hypoxia, and hyperoxia, resulting in mitochondrial dysfunction. mtROS production in the respiratory chain depends on the release of electrons out of the ETC. Electrons that leak out of the ETC react with oxygen to produce O^2-^, which detoxifies into H_2_O_2_ via SOD. Glutathione peroxidase (GPx) converts H_2_O_2_ to water. If unquenched, H_2_O_2_ can form the highly reactive OH^·^ by reacting with metal ions. Reduced MMP and mitochondrial calcium overload are important indicators of mitochondrial dysfunction, which has a profound influence on oxidative stress, inflammatory response, and apoptosis. mtDNA mutation and release impair mitochondrial respiratory chain, amplify mtROS, and accelerate mitochondrial dysfunction. Defective mitophagy results in the accumulation of damaged mitochondria. These abnormalities are able to cause oxidative stress, inflammasome activation, apoptosis, and senescence.

Mitochondrial homeostasis can be disrupted by environmental insults including tobacco smoke, air pollutants, inflammation, infection, hypoxia, and hyperoxia, resulting in mitochondrial dysfunction. This is characterized by increased mtROS, reduced MMP, mtDNA damage and release, and abnormal calcium signaling and storage. These abnormalities are able to cause oxidative stress, inflammasome activation, apoptosis, senescence, and metabolic reprogramming [15] (Figure 2). The role of mitochondria in disease progression is extensively investigated and suggests a direct link between mitochondrial dysfunction and the development of chronic respiratory diseases. Mitochondrial dysfunction is considered an important pathogenic mechanism of chronic respiratory diseases, including chronic obstructive pulmonary disease (COPD), pulmonary fibrosis, and asthma [2, 16, 17]. In this review, we discuss how mitochondrial dysfunction results in abnormal cellular processes and its role in the development of these chronic respiratory diseases. This not only enhances understanding of mechanisms of mitochondrial dysfunction underlying the pathogenesis of chronic respiratory diseases but also suggests potential therapeutic approaches for these diseases by targeting dysregulated mitochondria.

## 2. Mitochondrial Dysregulation in Pathological Processes

### 2.1. Abnormal mtROS Generation

Mitochondria are the major source of cellular ROS during OXPHOS. Electrons that leak out of the complex I (NADH dehydrogenase) oxidize oxygen to generate superoxide anion (O_2_^−^). O_2_^−^ can be dismutated into hydrogen peroxide (H_2_O_2_) by manganese superoxide dismutase (MnSOD), a mitochondrion-specific antioxidant. Glutathione peroxidase converts H_2_O_2_ to water. Meanwhile, H_2_O_2_ can form the highly reactive hydroxyl radical (OH^·^) by reacting with metal ions [18]. Hence, production of ROS in the respiratory chain is dependent on the release of electrons out of the ETC followed by the formation of these free radicals. Although the ETC is a major ROS producer in mitochondria, there are still other mitochondrial proteins involved in mtROS generation. These proteins include enzymes in the tricarboxylic acid cycle (i.e., aconitase, pyruvate dehydrogenase, and *α*-ketoglutarate dehydrogenase), inner mitochondrial membrane proteins (e.g., cytochrome P450 enzymes and glycerol-3-phosphate dehydrogenase), and outer mitochondrial membrane protein (e.g., cytochrome b5 reductase and monoamine oxidase) [19]. Under physiological conditions, low levels of mtROS serve as second messengers in coordinating biological and physiological processes [20, 21].

Abnormal mtROS has a causal role in mitochondrial dysfunction [22]. A variety of environmental toxins/oxidants and hyperoxia cause mtROS overproduction, which could damage mitochondrial protein, mitochondrial lipid membrane, and mtDNA [23]. Zorov et al. revealed that mtROS release causes the loss of MMP and increases mitochondrial permeability, called mitochondrial permeability transition (MPT) induction [24]. MPT induction alters the rigidity of the mitochondrial membrane and weakens protein-protein interactions which is necessary for the proper function of the respiratory chain [25]. Thus, the mtROS bursts are partly attributed to a block of the ETC caused by MPT induction [26]. Due to a lack of protective histones, mtDNA is susceptible to damage by mtROS, leading to mtDNA mutation. In turn, mtDNA mutation impairs the OXPHOS process further resulting in mtROS production [27–29]. These findings suggest vicious feedback between mtROS and mitochondrial dysfunction.

It has been shown that mtROS accumulation causes a range of cellular processes, including inflammation, apoptosis, and senescence [30]. For instance, Silvia et al. discovered ethanol-induced NOD-like receptor family pyrin domain-containing-3 (NLRP3) inflammasome activation upon mtROS generation. A mitochondrion-targeted antioxidant Mito-TEMPO abrogates mtROS release and reduces the upregulation of IL-1*β* and IL-18 induced by ethanol, effectively inhibiting NLRP3 activation [31]. Similar results are observed in the kidneys of patients with diabetic nephropathy and db/db mice; mtROS overproduction is accompanied by increases in NLRP3/IL-1*β* expression, which can be ameliorated by MitoQ, a mitochondria-targeted antioxidant [32]. In addition, mtROS increases mitochondrial membrane permeability. This leads to the escape of mtDNA into the cytoplasm, and mtDNA binds and activates the NLRP3 inflammasome [30, 33, 34]. Besides NLRP3 inflammasome, mtROS is able to activate a redox-sensitive mediator nuclear factor-*κ*B (NF-*κ*B), which regulates the transcription of various proinflammatory cytokines [35, 36].

Growing evidence indicates that mtROS accumulation modulates cellular apoptosis. Cytochrome C (Cyt c) release from mitochondria is a key event in initiating apoptosis [37]. Increased production of mtROS causes mitochondrial dysfunction including membrane potential loss and ATP depletion, which promotes the release of mitochondrial Cyt c, and activates caspase-9 and caspase-3 [38]. A recent study showed that Cyt c release is positively associated with the generation of mtROS [39]. A separate study discovered that free fatty acids result in elevated mtROS generation, promoting mitochondrial accumulation of p53 followed by recruiting proapoptotic factor Bax, initiating apoptotic events. In contrast, treatment with MitoQ inhibits mtROS generation and alleviates apoptosis in high fat-fed mice [40]. Similarly, cudraflavone C induces apoptosis in melanoma cells by increasing mtROS production, enhances the phosphorylation of MAPKs (p38, ERK, and JNK), and increases the expression of apoptotic proteins (Bax, Apaf-1, Cyt c, caspase-9, and caspase-3/7). Pretreatment with Mito-TEMPO alleviates MAPK activation, expression of apoptotic proteins, and apoptosis [41].

A large body of studies have been published to support the role of mitochondrial oxidant production in the senescence/aging process, identifying that the production of mtROS is the major determinant of senescence/aging [42, 43]. Overexpression of catalase targeted to mitochondria prevents cardiac aging [44]. The accumulation of mtROS and mitochondrial homeostasis imbalance trigger p53/p21 and/or p16/pRb pathways, leading to cellular senescence [45–47]. Indeed, mitochondrial dysfunction is considered an inducer of cellular senescence where mtROS is the most studied factor through persistent mtDNA damage. Due to the lack of histone, limited DNA repair capabilities, and proximity of mtDNA to the site of mtROS generation, mtDNA is sensitive to mtROS stimuli [48]. Excessive mtROS induces various types of mtDNA damage, including mtDNA mutation and decreased mtDNA copy number, which is thought to have a causal role in many age-related pathologies [27, 49]. In addition, excessive mtROS triggers the activation of the mitochondrial permeability transition pore (PTP). This further increases production and release of mtROS, which damages both mitochondrial and nuclear DNA, phospholipids, and proteins. PTP induction also releases matrix NAD that is hydrolyzed in the intermembrane space, thereby resulting in the depletion of cellular NAD, and this accelerates senescence [50, 51]. These results indicate that mtROS-induced cellular senescence mainly depends on mitochondrial oxidative damage, such as mtDNA mutations and mitochondrial membrane permeability. However, it is also possible that mtROS serves as signaling molecules to induce cellular senescence, independent of mitochondrial oxidative damage.

### 2.2. Reduced MMP

The MMP depends on ETC or the F1F0 ATP synthase system. It is an essential component in the process of energy storage during OXPHOS [52]. Under normal conditions, the ETC is the basic MMP producing system; the electron passes from complex I to complex IV and then generates MMP [53]. When the ETC function is abnormal, the F1F0 ATP synthase maintains MMP by reversing its action and hydrolyzing ATP. The ATP hydrolysis creates a direct electric current that is transferred from F1 to F0 where it is used to charge MMP [54]. MMP forms the transmembrane potential of hydrogen ions, which is harnessed to generate ATP [52]. Stabilization of MMP is the key determinant of mitochondrial performance, mitochondrial permeability, mitochondrial viability, nutrient import/output, and other important cellular functions [54].

Healthy mitochondria maintain high membrane potential, whereas the destruction of mitochondrial function can cause depolarization or a decrease in MMP [55]. Reduced MMP has been recognized as an important indicator of mitochondrial dysfunction and has a profound influence on inflammatory response and cellular apoptosis [56, 57]. Compared to controls, patients with systemic inflammatory response syndrome caused by sepsis, trauma, or others have a significant increase in platelet MMP depolarization [56]. Similar results are identified in contentious-flow left ventricular assist device patients with systemic inflammatory response syndrome; depolarization of MMP is significantly enhanced [58]. Consistently, age-related retinal inflammation is reduced by 670 nm light via increased MMP [59]. These results suggest that reduced MMP is associated with inflammation activation. Moreover, mitochondrial depolarization triggers inflammation, dependent on mitochondrial membrane permeability opening, which allows the release of mitochondrial components mtROS, mtDNA, or intermembrane space proteins into the cytosol [60]. MMP reduction-triggered inflammation activation may be more intricate, which still needs to be investigated.

The decrease in MMP can be triggered by the withdrawal of growth factors, deficiency of the extracellular glucose supply, blockage of respiration, or uncoupling of the inner membrane. Reduced MMP is a critical event in inducing cellular apoptosis [61, 62]. There is an evidence linking the decline in MMP to structural changes of mitochondria, including matrix condensation and cristal unraveling. These structural changes result in Cyt c release from the cristae to the intermembrane space and cytosol [57]. The research further discovers that when mitochondria are depolarized, matrix volume decreases and density increases, but when the mitochondria are energized, it is the opposite. This suggests that manipulation of mitochondrial depolarization alters mitochondrial configuration, thereby influencing apoptosis. Mitochondrial membrane depolarization could sensitize human granulosa tumor cells to induction of apoptosis [63]. In addition, when mitochondria are incubated at pH 6.4, oxidizable substrate-deprived, condensed mitochondria undergo decondensation of their matrix, as compared to incubation at physiological pH 7.4 [57]. Hence, the manipulation of the proton gradient across the mitochondrial membrane by acidifying the medium may be efficient for repairing MMP and controlling apoptosis.

### 2.3. Mitochondrial Calcium Dysregulation

The ER lumen is identified as the major intracellular calcium storage compartment. Mitochondria are closely linked to the ER, and approximately 20% of the mitochondrial surface is found in close proximity to the ER [64]. The close apposition between the ER and mitochondria facilitates calcium flux into the mitochondria. The ER calcium release machinery mainly involves in the inositol 1,4,5-trisphosphate (IP3) receptor [65]. The transferring of calcium into the mitochondrial intermembrane space is mediated by a voltage-dependent anion channel on the mitochondrial outer membrane at the ER-mitochondria contacts [13]. Calcium enters into the mitochondrial matrix as a key intracellular second messenger by binding and triggering changes in protein shape and charge, regulating proteins, enzymes, and transporters responsible for ATP synthesis [66]. Calcium has been shown to regulate the TCA cycle by activating three mitochondrial dehydrogenases, such as pyruvate dehydrogenase, isocitrate dehydrogenase, and *α*-ketoglutarate dehydrogenase, thereby controlling the rate of ATP synthesis [67]. More recently, some reports demonstrate that calcium could directly stimulate ATP production through activation of complex V and the F1F0 ATP synthase and promote electron transport via complex III of ETC [68, 69]. Mitochondrial calcium also modulates other cell functions, including MPT pore opening and Cyt c release [23, 70, 71].

Mitochondrial calcium balance is controlled by mitochondrial calcium uptake and release systems. There is a general consensus that mitochondrial calcium uptake is mediated by a macromolecular structure, the mitochondrial calcium uniporter (MCU) complex [72]. The MCU contains several components, including the MCU paralog MCUb, a family of related EF-hand-containing proteins (MICU1, MICU2, and MICU3), and EMRE, which composition appears to differ among various cell lines and tissues [73]. Calcium release depends on two different mechanisms, one mediated by a 2H^+^/Ca^2+^ antiporter (mHCX) expressed in most of the cells and the other by the control of the Na^+^/Ca^2+^ exchanger encoded by the NCLX gene [74, 75]. In addition, mitochondrial calcium homeostasis is also associated with the ER calcium balance. Existing evidence proposes that mitochondrial calcium disorder is possibly a result of ER calcium imbalance. Sarcoplasmic/endoplasmic reticulum Ca^2+^-ATPase (SERCA) is a pump that transports calcium ions from the cytoplasm into the ER. SERCA attenuates calcium overload and prevents activation of xanthine oxidase, resulting in decreased mtROS and improved mitochondrial quality control [76].

Mitochondrial calcium overload is closely associated with the production of oxidative stress [77, 78]. As shown by in vitro experiments, the addition of calcium to isolated rat heart mitochondria in the presence of antimycin A, a complex III inhibitor, induces ROS formation [79]. Inhibition of the activity of the MCU by an MCU inhibitor RU360 or small interfering RNA (siRNA) decreases mitochondrial calcium uptake, which renders the cells resistant to oxidative stress in in vitro experiments. In contrast, overexpression of the MCU causes higher sensitivity to the oxidative stress of cells [78, 80]. Silencing of NCLX expression decreases mitochondrial calcium release and aggravates high glucose-induced oxidative stress [81]. A recent study has shown that mitochondrial calcium overload triggers the net production of ROS through activation of mitochondrial PTP with the release of Cyt c, inhibition of respiratory chain, release of pyridine nucleotides, and loss of intramitochondrial glutathione necessary for the detoxification of peroxides [82].

NLRP3 inflammasome could be activated by calcium signaling [83]. Increased intracellular calcium concentration leads to calcium accumulation in the mitochondrial matrix via MCU leading to MMP loss, NLRP3 inflammasome activation, and IL-1*β* release [84]. Silencing of NCLX expression promotes NLRP3 inflammasome activation [81]. Rimessi et al. demonstrated that inhibition of MCU by the MCU inhibitor KB-R7943 could be beneficial for alleviating the *P. aeruginosa*-dependent inflammatory response in cystic fibrosis [85]. NLRP3 inflammasome activation depends on MCU, indicating that mitochondrial calcium is a novel molecular activator of NLRP3. Indeed, Lee et al. demonstrated that the murine calcium-sensing receptor activates NLRP3 inflammasome by increasing intracellular calcium and decreasing cyclic adenosine monophosphate (cAMP), suggesting the intimate relationship between calcium signaling and inflammasome activation. Several NLRP3 activators mobilize calcium, thereby promoting mitochondrial damage [86]. Thus, mitochondrial calcium overload induces activation of mitochondrial PTP as well as mtROS generation, which stimulates the release of mtDNA, leading to NLRP3 activation [87, 88].

Mitochondrial matrix calcium overload not only provokes the generation of oxidative stress but also causes the release of Cyt c, resulting in apoptosis [89–92]. For instance, an MCU inhibitor Ru360 significantly decreases amyloid-beta-induced microglial apoptosis, whereas apoptosis is induced by the MCU activator spermine [93]. Similarly, knockdown of endogenous MCU decreases mitochondrial calcium uptake and attenuates apoptosis induced by oxidative stress [80]. When large quantities of calcium are accumulated in the mitochondrial matrix, calcium interacts with cyclophilin D to trigger the opening of the mitochondrial PTP, leading to matrix swelling and outer mitochondrial membrane rupture, and Cyt c release. Moreover, a variety of proapoptotic factors converge on the PTP to control its calcium sensitivity, such as members of the B-cell lymphoma 2 (Bcl-2) family proteins [94, 95].

### 2.4. mtDNA

As previously mentioned, mtDNA is sensitive to mtROS stimuli. Damaged mtDNA can amplify mtROS and trigger oxidative stress through encoding deficient subunits for the respiratory chain, which accelerates the oxidative damage to mitochondrial function until cell death [96]. Therefore, it is possible that mtDNA damage contributes to the pathogenesis induced by oxidative stress [96]. Indeed, a positive relationship between ROS level and mutations in the D-loop region of mtDNA is observed in hepatocarcinoma tissues [97].

mtDNA serves as damage-associated molecular patterns (DAMPs) when released into cytosol or circulation. Lesions and release of mtDNA can trigger the inflammatory response through the Toll-like receptor 9 and cyclic GMP-AMP synthase- (cGAS-) stimulator of interferon genes (STING) [98–100]. For instance, matrix mtDNA release to cytosolic activates cGAS-STING signaling and type I interferon synthesis, which induces cell death-associated inflammation [101]. Damaged mtDNA escaping into the cytosol could activate ERK1/2, PI3K/Akt, tuberin, and mTOR via cGAS-STING, leading to increased expression of inflammatory mediators [102]. In contrast, STING knockdown or pretreatment with BAY11-7082 (an NF-*κ*B inhibitor) attenuates mtDNA-induced TNF-*α* and IL-6 expression in cultured Kupffer cells [103]. mtDNA activates NLRP3 inflammasome by mechanisms that involve calcium influx and mtROS generation [104]. Furthermore, the release of oxidized mtDNA into the cytosol, where it directly bounds to and activates the NLRP3 inflammasome, stimulates IL-1*β* secretion [105]. In addition, mtDNA damage activates NOD-like receptor family CARD domain-containing protein 4 inflammasome [106].

Damaged mtDNA has been implicated in cell survival and apoptosis [101, 107]. Ding et al. indicated that mtROS generation during a proinflammatory state induces mtDNA damage and enhances the expression of proprotein convertase subtilisin/kexin type 9 (PCSK9). Between damaged mtDNA and PCSK9 expression manifest a bidirectional cross-talk through p38 MAPK and mTOR activation. Simultaneously, elevated PCSK9 levels induce cell apoptosis by stimulating caspase-3 [108]. mtDNA damage and disordered calcium homeostasis are the main mechanisms for CdCl_2_-induced apoptosis [107]. Conversely, an increase in the mtDNA copy number via transfecting with an adeno-associated virus vector containing mitochondrial transcription factor A could protect hair cells and HEI-OC1 cells against drug-induced apoptosis. Activation of cytosolic cGAS-STING signaling pathway by mtDNA release could cause pyroptosis, a proinflammatory type of cell death [101, 109]. In addition, mitochondrial inner membrane permeabilization opening can occur during cell death following BAX/BAK-dependent mitochondrial outer membrane permeabilization. This enables the cytosolic release of mtDNA, and inner membrane permeabilization underpins the immunogenic effects of caspase-independent cell death [101]. Hence, matrix mtDNA release from the inner membrane plays a vital role in mitochondrial permeabilization-mediated cell death.

### 2.5. Dysregulated Mitophagy

Mitophagy is a selective and adaptive response and plays a critical role in surveilling mitochondrial quality and preventing the accumulation of dysfunctional mitochondria. Mammalian mitophagy is induced by two main pathways, specifically damage-induced mitophagy or developmental-induced mitophagy [110]. Damage-induced mitophagy is induced by two main proteins: (i) serine/threonine-protein kinase PINK1 that encodes the PTEN-induced putative kinase and (ii) Parkin (namely, PARK2) that is an E3 ubiquitin protein ligase [111]. Under normal conditions, PINK1 is targeted to the mitochondria, subsequently degraded by matrix processing peptidases and presenilin-associated rhomboid like. After the loss of MMP or the accumulation of misfolded proteins, PINK1 is stabilized on the outer mitochondrial membrane, promoting activation of autophagy recruitment machinery and triggering mitochondria degradation [112]. There are two different recruitment pathways regarding PINK1-mediated mitophagy. The canonical pathway is dominated by Parkin ubiquitination of mitochondrial protein. PINK1 is recruited on the outer mitochondrial membrane and phosphorylates ubiquitin to induce the recruitment and phosphorylation of E3 ubiquitin ligase Parkin, leading to the formation of autophagosomes. In this pathway, PINK1 has a small initiator role with the main function being to bring Parkin to the mitochondria. PINK1 can also recruit NDP52 and optineurin to mitochondria to activate mitophagy directly, independently of Parkin [113]. These are required to preserve the LC3-interacting region containing p62, which serves as an adaptor molecule to recruit autophagosome membranes in mitochondria. On the other hand, developmental process-induced mitophagy can be induced by the proapoptotic protein, Nip3-like protein X (Nix, also known as Bnip3L), and adenovirus E1B 19 kDa-interacting protein 3 (Bnip3), a member of the Bcl-2 family [114]. They prefer to eliminate mitochondrial population during differentiation, rather than remove unhealthy mitochondria [110]. FUN14 domain-containing protein 1 (FUNDC1) is a novel mitophagy receptor and governs mitochondrial turnover via the interaction with recruiting light chain 3 (LC3) to mitochondria [115]. FUNDC1-mediated mitophagy has been described in myocardial ischemia-reperfusion injury, acute kidney injury, and alcohol-related liver disease, attenuating hypoxia-related cardiomyocyte death and inhibiting the progression of diseases [115–117].

Dysregulated mitophagy has been involved in oxidative stress, inflammation, and apoptosis [118–120]. Resveratrol decreases oxidative stress by restoring mitophagy, while knocking down PINK1 reverses resveratrol-mediated ROS reduction [121]. Similarly, mitoquinone activates mitophagy through mitochondrial PINK1/Parkin/PHB2/LC3II pathways and attenuates oxidative stress and neuronal death after subarachnoid hemorrhage in rats [122]. Knockdown of Parkin increases H_2_O_2_-induced oxidative stress [123]. Autophagy/mitophagy-deficient cells with Bnip3 deletion display higher cellular ROS generation compared to the control group during myoblast differentiation. Decreased mitophagy results in the accumulation of damaged mitochondria with a greater propensity for apoptotic cell death signaling in the myoblast throughout differentiation [118].

Removal of damaged mitochondria and excessive ROS blocks the activation of mitochondrial dysfunction-induced inflammasomes and the release of inflammatory cytokines [124]. Both Parkin^−/−^ and PINK1^−/−^ mice have an increased inflammatory phenotype following exhaustive exercise [125, 126]. During contrast-induced acute kidney injury, mitochondrial damage, mtROS, and subsequent NLRP3 inflammasome activation are more severe in PINK1- or PARK2-deficient mice compared to the wild-type (WT) group [127]. Circulating mtDNA levels and ratios of mtDNA to nuclear DNA are higher in 40-week-old Parkin^−/−^ mice compared with WT mice, demonstrating that Parkin and PINK1 prevent inflammation by clearing damaged mitochondria [128]. Macrophage-specific p62 ablation reduces mitophagy but causes pronounced accumulation of damaged mitochondria and excessive IL-1*β*-dependent inflammation [129]. Upon LPS stimulation, knockdown of PINK1 via transfection with PINK1 siRNA in macrophages induces the accumulation of dysfunctional mitochondria compared with the control group.

The level of active caspase-3 increases in cells deficient of PINK1 during LPS stimulation [130]. Matrine represses mitophagy via blocking the PINK1/Parkin pathways, thereby inducing mitochondrial dysfunction and apoptosis in HepG2 cells. Reactivation of mitophagy by the overexpression of Parkin abolishes the proapoptotic effects of matrine on HepG2 cells [131]. Similarly, inhibition of mitophagy by PINK1 or Parkin deficiency increases silibinin-induced breast cancer cell apoptosis, suggesting that mitophagy induced by silibinin treatment serves as a cytoprotective effect, leading to reduction of apoptosis of cancer cells [132]. The roles of mitophagy in apoptosis are cell- and tissue-specific in cancer vs. noncancer tissues.

## 3. Mitochondrial Dysregulation in Chronic Respiratory Diseases

As aforementioned, abnormal mtROS generation, reduced MMP, mitochondrial calcium overload, mtDNA mutation, and dysregulated mitophagy play important roles in modulating oxidative stress, inflammation, apoptosis, and senescence. These cellular processes are associated with the development of chronic respiratory diseases, such as COPD, pulmonary fibrosis, and asthma [2, 16, 17]. Here, we discuss the role of mitochondrial dysfunction in the pathogenesis of these diseases.

### 3.1. COPD

COPD is one of the most common chronic diseases in the world. This disease encompasses emphysema, chronic bronchitis, and small airway obstruction, where cigarette smoking (CS) is a high-risk factor [133]. Although CS has been identified as the major risk factor for COPD, 15-20% of all smokers develop COPD, which may be attributed to genetic susceptibility [134]. Long-term CS exposure induces strong and persistent structural changes in mitochondria from COPD epithelium [135]. Here, we discuss the involvement of mitochondrial dysfunction in the pathogenesis of COPD in terms of mtROS, MMP, mtDNA, mitochondrial calcium, and mitophagy [136].

#### 3.1.1. mtROS

Increased mtROS and decreased MnSOD levels are observed in mitochondria isolated from bronchial biopsies from COPD patients compared to healthy never- and ex-smokers [137]. Similarly, human airway smooth muscle cells isolated from patients with COPD have reduced mitochondrial complex, ATP content, and basal and maximum respiration and increased mtROS compared with those from healthy control subjects [138]. Production of H_2_O_2_ by isolated mitochondria from the vastus lateralis is significantly higher in patients with COPD than control subjects with normal lung function, and complex III is identified as the main mitochondrial site of excess ROS production in skeletal muscle of patients with COPD [139]. CS extract (CSE) increases mitochondrial elongation/fragmentation and mtROS and reduces ATP levels in lung epithelial cells and fibroblasts [140]. Pulmonary vascular endothelial barrier injury and inflammation are important pathophysiological processes in CSE-induced COPD. Human umbilical vein endothelial cells treated with MitoQ protect against CSE-induced endothelial barrier injury and inflammation [141]. The iron-responsive element-binding protein 2 (IRP2), as a regulator of mitochondrial function, increases mitochondrial iron loading and levels of Cyt c oxidase, which leads to mitochondrial dysfunction and subsequent experimental COPD. Mice deficient in IRP2 are protected from CS-induced experimental COPD. In vitro, human airway epithelial cells deficient in IRP2 are protected from CSE-induced cell death and mtROS. In contrast, mitochondrial iron chelation alleviates CS-induced mtROS, bronchitis, and emphysema in mice with established COPD [142].

#### 3.1.2. MMP

Decreased MMP is observed in mitochondria isolated from bronchial biopsies from COPD patients compared to healthy never- and ex-smokers [137]. Human airway smooth muscle cells from patients with COPD also have reduced MMP compared with those from healthy control subjects [138]. When the epithelial cells are treated with 10% CSE, MMP and ATP production is significantly reduced, whereas ROS and apoptosis are elevated [143]. Compared with normal human bronchial epithelial cells, CS solution- and PM2.5-CS solution-treated cells show a significant loss of MMP, whereas PM2.5-CS solution-treated human bronchial epithelial cells than CS solution cells show a greater loss of MMP. Meanwhile, caspase activities increase significantly in CS solution-treated cells and increase more dramatically in PM2.5-CS-treated cells. This provides a new idea about the mechanism of PM2.5 on COPD, and inferred cigarette-inflamed airways are more sensitive to PM2.5 than normal airways [144]. In addition, compared with the control group, MMP and ATP levels in the quadriceps muscle are significantly reduced in CS-exposed rats, while apoptosis is increased. Compared with the model group, MMP and ATP levels increase significantly in the Bufei Jianpi and aminophylline groups, while apoptosis is lower. These findings suggest that Bufei Jianpi granules attenuate mitochondrial dysfunction via increasing MMP and ATP levels and subsequently alleviate cell apoptosis in peripheral muscles and the development of COPD [145].

#### 3.1.3. Mitochondrial Calcium

ER depletion of calcium storage is decreased in patients with COPD, and the calcium release from the ER is significantly decreased in epithelial cells from smokers (regardless of COPD status). Calcium release is clearly impaired in smokers with COPD, whereas smokers without COPD seem to be protected from the action of CS on ER calcium release. However, they have not identified the specific calcium pathway affected by COPD, and the changes of mitochondrial calcium remain unclear [146]. Intracellular calcium levels are increased in human lymphocytes in patients with COPD as compared to nonsmokers and smokers without COPD. H-DHPM, a novel calcium channel blocker, treated cells show a decrease in the intracellular calcium level as compared to the control cells and restore endothelial nitric oxide synthase expression in lymphocytes from COPD patients [147]. To date, the changes of mitochondrial calcium influx in the pathological progress of COPD are unclear, which needs to be further investigated.

#### 3.1.4. mtDNA

Zhang et al. evaluated the association of plasma mtDNA (p-mtDNA) with COPD severity and progression in the SPIROMICS cohort with 700 subjects [148]. They found that p-mtDNA levels have no significant differences between nonsmokers and ever smokers without COPD. Compared to nonsmokers and smokers without airflow obstruction, p-mtDNA levels are higher in mild or moderate COPD subjects. Notably, severe COPD participants have lower p-mtDNA levels compared with mild or moderate COPD participants. This indicates that mitochondrial dysfunction may play a role in the transition from mild to severe disease in COPD. In addition, the p-mtDNA levels have sex differences in subgroup analysis, a difference remaining significant after adjustment for age and smoking status [148]. Another study found that urine mtDNA and p-mtDNA available on the same patients did not linearly associate in the SPIROMICS cohort, suggesting that urine mtDNA is not simply a result of circulating p-mtDNA filtered by the kidney. This may have entirely different sources and represents distinct biological phenomena [149]. Specifically, urine mtDNA levels are measured and compared in the following four groups, including the never smokers, smokers without airflow obstruction, mild/moderate COPD, and severe COPD groups. Urine mtDNA levels are associated with increased respiratory symptom burden, especially among smokers without COPD. There are significant sex differences in urine mtDNA levels in patients with COPD. Urine mtDNA is only associated with worse spirometry in male patients with emphysema, while with worse respiratory symptoms in females only. These findings suggest that extracellular mtDNA levels may contribute to identifying distinct clinical phenotypes and underlying pathobiological differences in males versus females with COPD [149].

In addition, patients with asthma-COPD overlap syndrome have an increase in the mtDNA/nDNA ratio in the blood [150]. In mice, exposure to CS elevates the levels of DAMPs, reflected by an increase in mtDNA, high mobility group box-1, heat shock protein 70, and double-stranded DNA, and increases numbers of neutrophils in bronchoalveolar lavage (BAL) fluid, which is statistically reduced upon treatment with necrostatin-1. In vitro, CSE-exposed human bronchial epithelial cells show a significant increase in mtDNA, high mobility group box-1, heat shock protein 70, and double-stranded DNA release, and elevate in the percentage of necrotic cells. CS exposure induces necrosis of bronchial epithelial cells and subsequent DAMP release, triggering neutrophilic airway inflammation [151].

#### 3.1.5. Mitophagy

Both enhanced and impaired mitophagy have been implicated in COPD pathogenesis. Fibroblasts from patients with COPD show impaired mitophagy, which is associated with increased senescence [140]. Parkin protein levels are decreased in COPD lungs compared with non-COPD lungs. Reduced Parkin levels lead to the lack of mitophagy, accelerating cellular senescence in bronchial epithelial cells [152]. Parkin protein is the rate-limiting factor in PINK1-Parkin-mediated mitophagy. In comparison to WT mice, Parkin knockout mice have aggravated emphysematous changes, accumulation of damaged mitochondria, and oxidative modifications accompanied by accelerated cellular senescence, following CS exposure. Conversely, induced mitophagy by Parkin overexpression mitigates the progression of COPD [153]. In vitro, mitophagy plays a pivotal role in the removal of CS-induced mitochondrial dysregulation; silencing PINK1 and Parkin aggravates the development of COPD [152]. Impaired mitophagy aggravates CS stress-induced cellular senescence during the development of COPD [140]. These researches indicate that activating mitophagy prevents the pathological progression of COPD. However, Mizumura et al. reported that lung epithelial cells isolated from COPD patients display increased expression of PINK1. PINK1 deletion protects against CS-induced mitochondrial dysfunction, airspace enlargement, and mucociliary clearance in mice. Genetic deficiency of PINK1 and the mitochondrial division/mitophagy inhibitor Mdivi-1 protect against CS-induced necroptosis and mitochondrial dysfunction in vitro [154]. CS could increase Nix protein expression and induce mitophagy. Nix overexpression enhances mitophagy and aggravates mitochondrial dysfunction and cell injury, promoting the progression of COPD [155]. Similarly, FUNDC1 serves as another mitophagy receptor, which is highly expressed in CSE-treated human bronchial epithelial cell line and in vivo CS-induced COPD mouse models. Meanwhile, silencing FUNDC1 reduces levels of IL-6 and TNF-*α* and inhibits CSE-induced mitophagy, cell apoptosis, and the progression of COPD [156]. These findings show that mitophagy exhibits a complex role in the pathological processes in COPD. Activated mitophagy plays dual roles in COPD, which is often specific to a particular scenario. Therefore, we conjecture that mitophagy might ascertain the final fate of a cell depending on a complex interaction between several factors, including regulating intracellular ATP levels, initiating autophagic cell death, or triggering other types of cell death by modulating overlapping molecules.

### 3.2. Pulmonary Fibrosis

Pulmonary fibrosis is a devastating lung disease with a median survival of only 3 years after diagnosis. It is characterized by the accumulation of extracellular matrix proteins in the lung interstitium, leading to distortion of normal lung architecture [157]. Current therapeutic approaches have not been successful in improving disease outcomes [158]. Pulmonary fibrosis is associated with aging, and the median age to diagnose this disease is approximately 66 years [159–161]. Senescent lung fibroblasts from pulmonary fibrosis patients exhibit mitochondrial dysfunction, including disrupted cristae and a diminished capacity for oxidative phosphorylation [162]. Increased superoxide production by dysfunctional mitochondria accelerates senescence in lung fibroblasts by prolonging DNA damage response [163]. Thus, mitochondrial dysfunction plays a critical role in mediating cell senescence and promotes the development of pulmonary fibrosis. Here, we summarize the role played by mitochondrial dysfunction in the development of pulmonary fibrosis, including mtROS, MMP, mtDNA, mitochondrial calcium, and mitophagy.

#### 3.2.1. mtROS

mtROS generation is associated with the development of pulmonary fibrosis. Alveolar macrophages from patients with pulmonary fibrosis have significantly greater mitochondrial H_2_O_2_ produced in the membrane or mitochondrial fractions, while normal subjects show no difference in mitochondrial H_2_O_2_ production. Mitochondria isolated from bleomycin-exposed mice generate increased mitochondrial H_2_O_2_ than saline controls [164]. Compared to WT mice, crocidolite/bleomycin-exposed mitochondrial-targeted catalase (MCAT) mice exhibit reduced pulmonary fibrosis as measured by lung collagen levels and lung fibrosis score. In in vitro, alveolar type II (AT2) cells isolated from WT mice, mtROS production is significantly increased following asbestos exposure. Notably, compared to the WT group, AT2 cells from MCAT mice have significantly decreased levels of asbestos-induced mtROS production. Targeting mtROS levels may be an efficient therapeutic target for preventing pulmonary fibrosis [165]. In vitro, challenge of bronchial airway epithelial cells with bleomycin enhances mitochondrial O_2_^·-^ production. Treatment of bronchial airway epithelial cells with Mito-TEMPO, a specific scavenger of mtROS, decreases mitochondrial O_2_^·-^, mtDNA damage, and apoptosis [166]. Increased mtROS production leads to mtDNA damage and apoptosis of alveolar epithelial cells, which are necessary for the development of pulmonary fibrosis [167]. Similarly, TGF-*β*1 stimulation of alveolar epithelial cells results in mtROS production, and this is reduced by Mito-TEMPO treatment [168].

#### 3.2.2. MMP

Loss of MMP is an important mechanism of the development of pulmonary fibrosis. Lung tissue cells isolated from paraquat-exposed rats have a significant loss of MMP compared with saline controls, while treatment with cyclosporine A, an inhibitor of mitophagy, significantly improves the paraquat-decreased MMP in the lung cells of rats [169]. The change in the MMP has been thought to be viewed as an early event of mitophagy. In vitro, macrophage treatment with bleomycin shows a loss in MMP in a dose-dependent manner [164]. Similarly, A549 cells display increased mitochondrial depolarization following bleomycin treatment [168]. This suggests that reduced MMP participates in the pathogenesis of pulmonary fibrosis.

#### 3.2.3. Mitochondrial Calcium

Alveolar macrophages are the predominant cells in BAL fluid from normal participants and patients with asbestosis. Compared with control subjects, patients with asbestosis have higher levels of mitochondrial calcium [170]. Similar findings have further demonstrated that MCU gene and protein expression are significantly higher in mitochondria of lung macrophages from subjects with pulmonary fibrosis compared to control subjects. In vivo, lung macrophages from bleomycin-injured mice have increased MCU expression. Mitochondrial calcium is markedly reduced in lung macrophages from bleomycin-exposed mice transfected with dominant negative MCU compared to WT controls [171]. Furthermore, these mice are protective from bleomycin-induced pulmonary fibrosis. In vitro, mouse alveolar macrophage cells are transfected with scrambled or MCU siRNA and exposed to asbestos. After asbestos exposure, mitochondrial calcium in MCU-silenced cells is significantly reduced to levels similar to that in unexposed cells. These findings suggest that the increased activity of MCU along with the overload of mitochondrial calcium in lung macrophages promotes the pathogenesis of pulmonary fibrosis [170]. Hence, MCU-mediated imbalanced calcium in mitochondria contributes to fibrotic repair after lung injury.

#### 3.2.4. mtDNA

Compared to controls, BAL fluid from pulmonary fibrosis subjects exhibits a robust increase in extracellular mtDNA. Likewise, plasma obtained from patients with pulmonary fibrosis shows substantial increases in mtDNA compared with control subjects. Plasma mtDNA concentrations are obtained prior to the initiation of antifibrotic agent pirfenidone therapy and three months after starting therapy. Compared to nonresponders, plasma mtDNA numbers are increased, whereas responders experience a reduction in mtDNA numbers. Excessive plasma mtDNA is predictive of all-cause mortality by a 43-month longitudinal follow-up. These findings indicate that plasma mtDNA measurements might be a useful predictor of treatment efficacy and clinical outcomes in pulmonary fibrosis [172, 173].

Sirtuin 3 (SIRT3) primarily localizes in mitochondria and could regulate acetylation of mitochondrial proteins MnSOD and 8-oxoguanine glycosylase, a critical role in the repair of mtDNA oxidative damage. In vivo, compared with WT controls, SIRT3-knockout mice show exacerbated fibrosis after bleomycin exposure. Increased pulmonary fibrosis is associated with decreased levels of 8-oxoguanine glycosylase and concomitant accumulation of oxidized guanine and increased mtDNA damage. Inversely, the transgenic mice with whole-body SIRT3 overexpression are protective against bleomycin-induced mtDNA damage and pulmonary fibrosis. In vitro, TGF-*β*1 treatment decreases the expression of endogenous SIRT3, subsequently increasing mtDNA damage. Conversely, overexpression of SIRT3 by adenovirus-mediated transduction reverses the effects of TGF-*β*1 on mtDNA damage and suppresses TGF-*β*1-induced myofibroblast differentiation [174, 175].

#### 3.2.5. Mitophagy

Alveolar AT2 cells from pulmonary fibrosis patients exhibit accumulation of dysfunctional mitochondria and impaired autophagy [159]. In vivo, PINK1-deficient mice develop similarly dysmorphic, dysfunctional mitochondria in the AT2 cells and are susceptible to apoptosis and development of pulmonary fibrosis [159]. Similarly, PARK2-knockout mice show enhanced pulmonary fibrosis after bleomycin exposure. This is associated with increased mtROS production and activation of the platelet-derived growth factor receptor (PDGFR)/PI3K/AKT signaling pathway, resulting in increased myofibroblast differentiation and proliferation for fibrotic foci formation [176]. Pirfenidone, an FDA-approved antipulmonary fibrosis drug, triggers mitophagy activation by enhancing PARK2 expression. PARK2 knockdown mice display increased pulmonary fibrosis, and this is efficiently alleviated by pirfenidone [177]. Thyroid hormone improves mitochondrial bioenergetics, promotes mitochondrial biogenesis, and attenuates mitochondria-regulated apoptosis in alveolar epithelial cells. Intriguingly, thyroid hormone does not alleviate the pathological process of pulmonary fibrosis in PINK1-knockout mice [178]. In vitro, knockdown of PINK1 expression in lung epithelial cells results in mitochondria depolarization and expression of profibrotic factors [159]. These results suggest that insufficient mitophagy aggravates the accumulation of dysregulated mitochondria, leading to the development of pulmonary fibrosis. This is in contrast to the findings that mitophagy is required for apoptosis resistance, which activation accelerates the development of pulmonary fibrosis. Larson-Casey et al. investigated that mitochondria in alveolar macrophages isolated from pulmonary fibrosis patients have higher expression of PINK1, Parkin, and LC3-II compared to control subjects. Likewise, WT mice exposed to bleomycin have enhanced PINK1 and Parkin expression in isolated mitochondria and increased LC3-II in alveolar macrophages. Alveolar macrophages from mice exposed to rapamycin, an autophagy/mitophagy activator, have increased levels of PINK1, Parkin, and p62, as well as LC3-II. Meanwhile, pulmonary fibrosis development is aggravated by rapamycin [164]. In contrast, cyclosporine A serves as an inhibitor of mitophagy, and its treatment mitigates the paraquat-treated collagen synthesis in A549 cells. This is attenuated by PINK1 overexpression [169]. The opposite results may ascribe that impaired mitophagy induces oxidative stress injury, but excessive mitophagy prevents apoptosis in a cell-specific manner. Further studies are warranted to investigate the role of FUNDC1, Nix, and Bnip3 in pulmonary fibrosis in terms of mitophagy.

### 3.3. Asthma

Asthma is a common condition due to chronic inflammation of the lower respiratory tract. Chronic psychosocial stress and environmental exposure such as allergens and air pollutants have been linked to the risk of asthma [179]. Pharmacologic therapies are often not effective in older asthmatic patients and may have more side effects [180]. Cellular, molecular, and animal model studies have revealed that mitochondrial dysregulation is recognized as a key mediator for asthma [181]. A separate study shows that increased bronchial smooth muscle mass in nonsevere asthmatics is associated with exacerbations and worse asthma control in 34 never smoker subjects with nonsevere asthma and 56 nonsevere and 19 severe asthmatics. Meanwhile, the number and the density of mitochondria in bronchial smooth muscle cells are positively correlated to the bronchial smooth muscle area in a subgroup of nonsevere asthmatics. The research further reveals the importance of mitochondria in the progression of asthma [182]. Long-term administration of corticosteroids induces mitochondrial dysfunction and oxidative damage of mitochondria and nuclear DNAs in skeletal muscles [183, 184]. It remains unknown whether the side effects of long-term corticosteroid therapy in asthma are related to mitochondrial dysfunction.

#### 3.3.1. mtROS

MMP, mtDNA, and mtROS participate in intercellular communication within the airways of human subjects with asthma [185]. mtROS production is increased in the venous blood of severe asthmatic patients compared with the control group [186]. In the ovalbumin-induced murine asthma model, mtROS production is increased in comparison with saline control. Mito-TEMPO significantly attenuates mtROS, TGF-*β*, and collagen deposition in ovalbumin-challenged mice. In vitro, human airway epithelial cells treated with IL-13 have an increase in mtROS and collagen expression, and this is attenuated by Mito-TEMPO treatment [187].

#### 3.3.2. MMP

Human peripheral blood eosinophils are purified from self-reported allergic asthma or healthy donors. MMP is reduced in eosinophils from allergic asthma donors compared with healthy donors [188]. Compared to the control group, the respiratory control ratio of mitochondria and MMP is significantly lower in the rat asthma models [189]. A separate study discovers that fibroblasts from severe asthmatics have reduced MMP and metabolic activity and increased expression of mitophagy genes, PINK1 and Parkin [190]. Whether rectifying or increasing MMP protects against asthma remains unclear.

#### 3.3.3. Mitochondrial Calcium

Compared to control subjects, mitochondrial mass and oxygen consumption are higher in the bronchial smooth muscle from asthmatic subjects, which is associated with enhanced extracellular calcium influx and mitochondrial biogenesis. This may promote the proliferation of bronchial smooth muscle cells, leading to airway remodeling [191]. A calcium channel blocker gallopamil decreases the proliferation of bronchial smooth muscle cells from patients with severe asthma. Gallopamil treatment for 12 months reduces bronchial smooth muscle remodeling and prevents the occurrence of asthma exacerbations [192]. In the ovalbumin model of allergic asthma, MCU knockdown mice show decreased apoptosis within the large airway epithelial cells compared to WT mice. Expression of the tight junction protein zona occludens-1 is not significantly changed in MCU knockdown mice. In contrast, ovalbumin-treated WT mice have significantly reduced zona occludens-1 staining within the airway epithelia. In primary human respiratory epithelial cells, MCU deficiency suppresses mitochondrial calcium uptake and ROS production, maintains MMP, and protects against apoptosis in response to the pleiotropic Th2 cytokine IL-13 [193]. Additionally, allergen-induced ROS generation activates calcium/calmodulin-dependent protein kinase II (CaMKII) that plays a role in downstream regulation of autophagy/mitophagy, resulting in hyperresponsiveness, ROS generation, and Th2-associated lung inflammation. Mitochondrial CaMKII inhibition mitigates allergen-induced autophagy/mitophagy, mitochondrial dysfunction, and cytokine production in bronchial epithelial cells and subsequent airway hyperreactivity, inflammation, and asthma [194].

#### 3.3.4. mtDNA

Through analysis of 16,158 mitochondrial single nucleotide polymorphisms, different sex-specific locations in the mitochondrial genome are observed in a human cohort with 372 asthmatic children (aged 5 to 18 years, 35% female and 65% male) and 395 healthy children (aged 5 to 18 years, 44% female and 56% male) [195]. One of the genetic causes of asthma in girls could be a dysfunction of the MT-ND2 and MT-RNR2 genes. In boys, variants in the CYB gene provoke changes in ROS production and could be causative of asthma [195]. Carpagnano et al. enrolled fifty-three patients with severe asthma, 11 patients with mild-moderate asthma, and 12 healthy subjects and measure the content of mtDNA and nuclear DNA (nDNA) in exhaled breath condensate. Higher exhaled mtDNA/nDNA is observed in severe asthmatics, respectively, compared to mild-moderate ones and to healthy controls [196].

To establish an in vivo model of asthma, WT and Parkin deficiency mice are intranasally inoculated with IL-13 or house dust mites. WT mice show significantly increased numbers of both neutrophils and eosinophils in BAL fluid following IL-13 treatment than Parkin deficiency mice. Treatment with IL-13 also increases mtDNA release and neutrophil chemokine levels in BAL fluid of WT mice compared with Parkin deficiency mice. In vitro, Parkin deficiency in IL-13-stimulated human tracheobronchial epithelial cells inhibits mtDNA release. This suggests a link of mitophagy, mtDNA, and inflammation in the pathogenesis of asthma [197]. Increased accumulation of cytosolic double-stranded DNA is observed in the airway epithelium of ovalbumin- or house dust mite-exposed mice and in human bronchial epithelial cells treated with IL-33. Interestingly, Mito-TEMPO could reduce IL-33-induced cytoplasmic double-stranded DNA accumulation in human bronchial epithelial cells, which is possibly associated with suppressing the release of mtDNA into the cytosol [198]. Altogether, mtDNA damage or release plays important roles in the pathogenesis of asthma.

#### 3.3.5. Mitophagy

PINK1/Parkin-mediated mitophagy is enhanced from severe asthmatic patients compared to healthy controls by bioinformatics analysis [190]. Brushed bronchial epithelial cells isolated from patients with asthma have significantly higher Parkin mRNA levels than healthy controls [197]. Additionally, Bnip3 expression is increased in airway smooth muscle cells from asthmatic donors compared to that from control donors. Silencing Bnip3 in human airway smooth muscle cells alleviates cell adhesion, migration, and proliferation and suppresses the development of asthma [199]. Moreover, there is a significant upregulation in the expression of LC3B and ATG5 in the lung tissues of asthmatic patients compared to healthy controls. In vivo, a cockroach extract-established asthmatic mouse model also shows significantly increased expression of LC3B and ATG5 in the lung tissues. In vitro, human bronchial epithelial cells show induction of mitophagy following cockroach allergen than controls. These results indicate that excessive activation of mitophagy plays an important mechanism in developing asthma [194]. Further study is required to determine the role of FUNDC1 and Nix in modulating mitophagy during the development of asthma.

## 4. Conclusion and Future Directions

**Figure 3:**
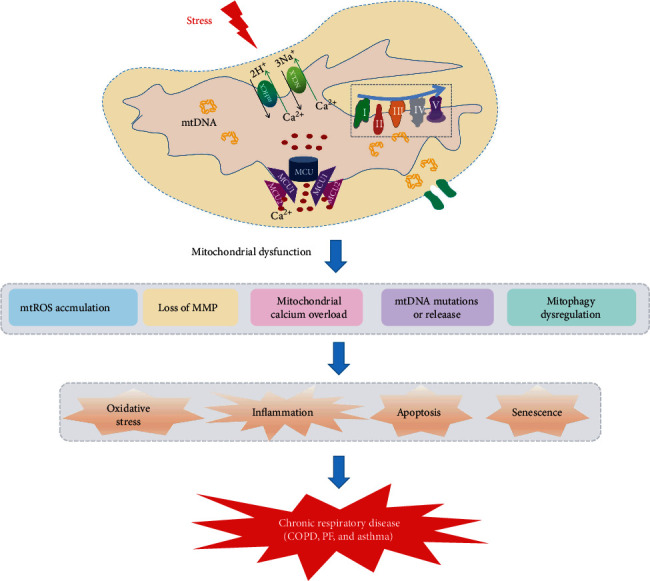
Mitochondrial dysfunction contributes to the development of chronic respiratory diseases. Mitochondrial dysfunction accompanies increased mtROS, reduced MMP, mitochondrial calcium overload, mtDNA damage and release, and abnormal mitophagy. This could cause a series of cellular processes, including oxidative stress, inflammasome activation, apoptosis, and senescence. All these cellular processes participate in the pathogenesis and progression of chronic respiratory diseases, including COPD, pulmonary fibrosis (PF), and asthma.

Although mitochondria are indispensable for energy metabolism and play important roles in modulating cell signaling and maintaining cell function and tissue homeostasis. Normal mitochondria are sustained by stabilization of MMP, balance of mitochondrial calcium, integrity of mtDNA, and timely clearance of damaged mitochondria via mitophagy. Mitochondrial dysfunction participates in the pathogenesis of COPD, pulmonary fibrosis, and asthma via mtROS overproduction, mtDNA damage and release, reduced MMP, and abnormal mitophagy (Figure 3). Targeting dysfunctional mitochondria could be a promising approach to prevent or halt the development of these chronic lung diseases.

Mitochondria are dynamic through fission and fusion. Quantitative imaging-based assessment of mitochondrial morphology and dynamics using the full 3D mitochondrial network with an extension to 4D analysis can provide valuable insights into lung cellular physiology and pathophysiology of these chronic lung diseases [200]. Moreover, there are many different cells in the lung. With the progress of technologies, single-cell RNA sequencing may provide directions on mitochondrial dysfunction in a cell-specific manner. A recent report showed that somatic mutations in mtDNA can be used as natural genetic barcodes to explore cellular states and clonal dynamics using single-cell sequencing technologies [201]. This has three key advantages, including being highly scalable, directly applicable to human tissues, and combined with assays to profile a cell's state at the chromatin or transcriptome level. To date, using single-cell RNA sequencing analysis to investigate the mechanism of mitochondrial dysfunction for chronic respiratory diseases has not been reported. In addition, using a newly developed digital spatial profiling platform (NanoString nCounter and GeoMX platforms), the temporal and spatial heterogeneity of mitochondrial biology and dysfunction could be dissected in these diseases. All those may open up novel avenues to further explore the pathogenesis progress of these diseases.
